# Profiles of lipids, blood pressure and weight changes among premenopausal Chinese breast cancer patients after adjuvant chemotherapy

**DOI:** 10.1186/s12905-017-0409-8

**Published:** 2017-07-27

**Authors:** Winnie Yeo, Frankie K. F. Mo, Elizabeth Pang, Joyce J. S. Suen, Jane Koh, Herbert H. F. Loong, Christopher C. H. Yip, Rita Y. W. Ng, Claudia H. W. Yip, Nelson L. S. Tang, Giok S. Liem

**Affiliations:** 1 Department of Clinical Oncology, Prince of Wales Hospital, The Chinese University of Hong Kong, Shatin, New Territories Hong Kong SAR; 2 State Key Laboratory in Oncology in South China, Prince of Wales Hospital, The Chinese University of Hong Kong, Shatin, New Territories Hong Kong SAR; 3 Department of Chemical Pathology, Prince of Wales Hospital, The Chinese University of Hong Kong, Shatin, New Territories Hong Kong SAR; 4 Li Ka Shing Institute of Health Sciences, Faculty of Medicine, Prince of Wales Hospital, The Chinese University of Hong Kong, Shatin, New Territories Hong Kong SAR

**Keywords:** Cytotoxic, Cholesterols, Dyslipidemia, Hypertension, Obesity

## Abstract

**Background:**

Adjuvant chemotherapy improves outcome of patients with early breast cancer. However, chemotherapy may be associated with long term toxicities. In this retrospective cohort study, the objectives were to determine body weight, body mass index (BMI), blood pressure and fasting lipids levels of young premenopausal Chinese breast cancer patients after adjuvant chemotherapy. Potential factors associated with these parameters were identified.

**Methods:**

Eligibility criteria include premenopausal Chinese patients who were diagnosed to have stage I-III breast cancer within 3–10 years, age < 45 and having received adjuvant chemotherapy at the time of breast cancer diagnosis. Information at initial breast cancer diagnosis were retrieved from patients’ medical records and include age at diagnosis, tumor characteristics, anti-cancer treatments, blood pressure and body weight and height. At study entry, all patients had additional background demographics collected, as well as blood pressure, body weight and fasting serum lipid profiles measured. Incidence of chemotherapy-related amenorrhoea (CRA) and menopause were determined. Factors associated with weight gain, hypertension and dyslipidaemias were analyzed.

**Results:**

Two hundred and eighty patients were studied. The median age at breast cancer diagnosis was 41 years (range: 24–45). The median time from breast cancer diagnosis to study entry was 5.0 years. The median age at study entry was 46.5 years (range: 28–54). 91.1% developed CRA; 48.9% had become menopausal and 10% were peri-menopausal. Between initial breast cancer diagnosis and the time of study entry, the median weight gain was 1.8 kg; 63.2% gained weight by >2%; 52.1% were overweight/obese; 30.7% had hypertension. Abnormal total-cholesterol and LDL-cholesterol occurred in 34.3% and 56.1% respectively. On multivariate analyses, older age was associated with reduced risk while occurrence of CRA and having received taxane-containing regimens were associated with increased risk of weight gain. Oestrogen-receptor positivity was associated with reduced risk while overweight/obese statuses were associated with increased risk of hypertension. Use of tamoxifen was associated with reduced risk of abnormal LDL-cholesterol. Weight gain, overweight/obese, older age, progression to post/peri-menopausal status at study entry, having received corticosteroid premedication before adjuvant chemotherapy and having received taxane-containing adjuvant chemotherapy were associated with increased risk of dyslipidaemias.

**Conclusion:**

Among young premenopausal Chinese breast cancer patients who had received adjuvant chemotherapy, the current study has revealed that although there was only a median weight gain of 1.8 kg, there was a nearly 60% increase in abnormal BMI. Further, a significant proportion of patients were detected to have hypertension and dyslipidaemias. Interventional studies with lifestyle modifications are warranted.

**Electronic supplementary material:**

The online version of this article (doi:10.1186/s12905-017-0409-8) contains supplementary material, which is available to authorized users.

## Background

In China, breast cancer is the most common female malignancy, with an age standardized rate of 27.96 per 100,000 population [1]; the corresponding figure is relatively higher in Hong Kong at 64.6 per 100,000 population, with over 80% of newly diagnosed breast cancer patients having diagnosed with early stage disease [2]. For women with early stage breast cancer who have undergone curative surgery, adjuvant therapies have shown to improve disease-free and overall survival. Anticancer treatment, especially cytotoxic chemotherapy, is associated with immediate as well as long-term toxicities, which may affect the quality of life and well-being of cancer survivors [3, 4]. Some of the chemotherapy-related long term toxicities include chemotherapy-related amenorrhoea (CRA) and early menopause, as well as anthropometric and metabolic changes that could lead to increased cardiovascular risk [5, 6].

Weight gain is a common occurrence in women diagnosed with breast cancer [7–10]. Patients who underwent adjuvant chemotherapy had reportedly gained more weight than those treated with hormonal or radio- therapies [9–12]; such difference continued to be observed over years, and a return to initial weight following weight gain was infrequent [9]. With old chemotherapeutic regimens, studies in the UK and the US had indicated that women who underwent chemotherapy gained an average of 2.5–6.7 kg in weight, although gains in excess of 10 kg had not been uncommon [13, 14]. Using contemporary chemotherapy regimens that included taxanes as well as anthracyclines, a French study reported a mean weight gain of 3.9 kg 1 year after treatment [15]. Apart from weight gain and obesity which is associated with detrimental effect on health profiles, anti-cancer drugs, specifically the anthracyclines and anti-human epidermal growth factor receptor 2 (HER2) agents that are commonly used in breast cancer, could cause cardiotoxicity and may further increase the cardiovascular risk of the patients concerned [16]. While change in menopausal status and increased body weight have been well-linked to the use of adjuvant chemotherapy, the association of these changes with cardiovascular comorbidities, specifically, aspects on blood pressure and lipids profiles, are less well-known.

The objectives of this retrospective cohort study on young premenopausal Chinese women with early breast cancer in Hong Kong were to (1) determine the body weight, body mass index (BMI), blood pressure and fasting lipids levels of young premenopausal breast cancer patients after adjuvant chemotherapy; and (2) identify potential factors associated with these parameters.

## Methods

Between September 2008 and February 2011, eligible breast cancer patients who were attending the breast cancer follow-up clinic of the Prince of Wales Hospital were approached for study enrolment. Eligibility criteria included female of Chinese ethnicity, a history of stage I-III breast cancer within 3–10 years, premenopausal, age younger than 45 years and having received adjuvant chemotherapy at the time of breast cancer diagnosis. Patients were excluded if they had evidence of disease recurrence. Patients who received ovarian ablation as part of the endocrine therapy or had hysterectomy prior to breast cancer diagnosis were also excluded. Eligible patients identified during their follow up visits were consented for the study. The study was approved by the Joint CUHK-NTEC Clinical Research Ethics Committee of the Chinese University of Hong Kong and Hong Kong Hospital Authority.

### Data collection

Clinical details at the time of breast cancer diagnosis were retrieved from individual patient’s medical records. These included patient’s age at breast cancer diagnosis, cancer characteristics (stage, oetrogen receptors [ER], progesterone receptors [PR] and HER2 status of the breast tumour) and information on treatment she received for her breast cancer (type of breast surgery, details of adjuvant radiotherapy, chemotherapy, tamoxifen and trastuzumab, and history of corticosteroid premedication during chemotherapy). Individual patient’s blood pressure, body height and weight, measured by clinic staff at the time of diagnosis, were also retrieved from the medical records.

At study entry, patients’ demographics (education level, employment history, level of family income, smoking and alcohol history, family history of breast cancer and number of live births before breast cancer diagnosis) were collected. Each patient had blood pressure and body weight measured by a research assistant. Each patient was asked to complete a study questionnaire in which they recalled their menstruation history with the assistance of a research assistant within the same hospital visit. In the questionnaire, patients were asked in details on the following: [1] last menstrual period (LMP) before commencement of chemotherapy; [2] after commencement of chemotherapy, presence or absence of a period of amenorrhoea with dates; [3] in case of occurrence of amenorrhoea, whether there was subsequent return of menstruation with dates; [4] for patients who had resumption of menstruation, any subsequent amenorrhoea with dates. For patients who did not experience amenorrhoea during chemotherapy, the subsequent menstrual history was further collected similarly. LMP prior to study entry was determined for each patient. Fasting blood was taken for lipid profiles, which included total-cholesterol, low density lipoprotein (LDL-) cholesterol, high density lipoprotein (HDL-) cholesterol and triglyceride levels.

### Definitions

CRA was defined as amenorrhoea for ≥3 months during and within 12 months after the completion of adjuvant chemotherapy [17]. Menopause was defined in line with World Health organization (WHO) criteria as 12 months of amenorrhoea with last menstrual period (LMP) ≥12 months after chemotherapy and before study entry [18].

At study entry, weight gain was defined as an increase in body weight of >2% when compared to the weight at breast cancer diagnosis [13], prior to the start of adjuvant therapies. According to the WHO criteria for Asians, BMI categories of underweight, normal weight, overweight and obesity were defined as <18.5, 18.5- < 23, 23- < 25, and >/= 25.0 kg/m2 respectively [19].

Based on criteria of International Society of Hypertension, hypertension was defined as rise in systolic blood pressure (SBP) to >/=140 mmHg, and/or increase in diastolic blood pressure (DBP) to >/=90 mmHg [20]. According to recommendation of the US National Cholesterol Education Program, dyslipidaemia was defined as fasting serum total-cholesterol >/=5.2 mmol/L, LDL-cholesterol >/=2.6 mmol/L, HDL-cholesterol </=1.0 mmol/L, and/or triglyceride >/=1.7 mmol/l [21].

### Statistical analysis

Statistical analysis was performed by SAS version 9.3. Continuous variables were expressed as means with standard deviation or median with range as appropriate. Baseline continuous variables were compared by Student’s t-test or Mann Whitney U test as appropriate, and categorical variables were compared by Chi-square test. All statistical tests were two-sided, and *p* values <0.05 were regarded as significant.

Univariate logistic regression was performed to identify any potential factors associated with weight gain, hypertension and dyslipidaemias post-chemotherapy. Stepwise multivariate logistic regression analysis that included significant factors was conducted.

## Results

In total, 300 breast cancer patients were approached for study entry, 14 of them declined participation. As a result, 286 patients were consented to participate in this study. Two patients failed to meet inclusion criteria as they received neo-adjuvant therapy for their stage IIIb breast cancers, four patients withdrew with the reason that they didn’t have time to perform the blood tests after consent. As a result, 280 eligible patients entered the study. Table 1 shows the patients’ background demographics, tumour characteristics, and anti-cancer treatments received after breast cancer diagnosis. With regards to educational level, majority (67%) of the patients received secondary school education, 17% had primary school education and 16% had tertiary or higher level of education. On employment, 45% were on full time employment, 16% had part-time employment and the remaining 38% were either unemployed or had retired. With respect to family income, 9.6% of patients had monthly family income less than HK$5000, 54% had income of HK$5000–25,000, 30% had income of HK$25000–50,000 and 7% had income over HK$50,000.

**Table 1:** Patients’ background demographic and clinical characteristics at the time of breast cancer diagnosis (*n* = 280)

	No. of patients	%
Age at breast cancer diagnosis		
≤ 35	41	14.6
36–40	82	29.3
41–45	157	56.1
Height- median; range (cm)	159 (141–175)	
Weight- median; range (kg)	54.6 (39.0–89.0)	
BMI at diagnosis (according to HK BMI)		
Underweight (<18.5)	33	11.8
Normal (18.5–22.9)	155	55.4
Overweight (23.0–24.9)	46	16.4
Obese (>25)	46	16.4
1 or more children born before breast cancer diagnosis	205	73.2
Ever smoking	10	3.6
Ever excessive alcohol intake (> 2 units/day)	1	0.4
Education		
Primary	47	16.9
Secondary	187	67.0
Tertiary	27	9.7
Higher qualification	18	6.5
Employment		
Retired	3	1.1
Unemployed	105	37.5
Full Time	127	45.3
Part Time	45	16.1
Family income		
< HK$5000	27	9.6
HK$5000–25,000	150	53.6
HK$25000–50,000	83	29.6
HK$50,000	20	7.2
1st degree relative with breast cancer	17	6.1
T stage		
T1	140	50.0
T2	133	47.5
T3	7	2.5
T4	0	0
Nodal Status- positive	114	40.7
TNM staging:		
Stage I	88	31.4
Stage II	165	58.9
Stage IIIa	27	9.6
ER positive	203	72.5
PR positive	187	66.8
HER2 over-expression	47	16.8
Breast surgery:		
Lumpectomy	95	33.9
Mastectomy	185	66.1
Axillary lymph node dissection	276	98.6
Received adjuvant radiotherapy	186	66.4
Adjuvant chemotherapy regimen:		
Non-anthracycline and non-taxane containing	17	6.1
Anthracycline-containing	184	65.7
Taxane-containing	5	1.8
Anthracycline- and taxane-containing	74	26.4
Duration of adjuvant chemotherapy >64 days	191	68.2
Received corticosteroid premedication during chemotherapy	258	92.1
Received adjuvant tamoxifen	214	76.4
Received adjuvant trastuzumab	8	2.9
Use of traditional Chinese medicine since diagnosis	83	29.6

The median age at breast cancer diagnosis was 41 years (range: 24–45). Eighty-eight had Stage I, 165 had stage II and 27 had Stage III breast cancer; 73% were ER-positive, 67% were PR-positive and 17% were HER2 positive. Adjuvant chemotherapy regimens included anthracycline-containing (65.7%), anthracycline-taxane containing (26.4%), taxane containing (1.8%) and non-anthracycline/non-taxane containing (6.1%). Two hundred and fourteen patients also received adjuvant tamoxifen. The median time from breast cancer diagnosis to study entry was 5.04 years (range: 2.96–9.94). The median age at study entry was 46.5 years (range: 28–54); eight were ≤35 years, 26 were aged 36–40, 76 were aged 41–45, 146 were aged 46–50 and 24 were aged >50. At the time of the study, 115 patients were still on adjuvant tamoxifen therapy.

Two hundred and fifty-five (91.1%) had experienced CRA. At study entry, 137 patients were postmenopausal and 28 were peri-menopausal. The median age of menopause was 44 years (range: 34–52). Details of the menstrual history of all patients were described in a previous report [14].

### Body weight and BMI at breast cancer diagnosis and at study entry

The median weight of patients at breast cancer diagnosis was 54.6 kg (range: 39.0–89.0), this increased by 1.8 kg to 56.4 kg (range: 39.5–92.6) at study entry. At study entry, 177 patients (63.2%) had gained weight by >2%, including 121 who gained >/=5%.

At study entry, 3.9% were underweight, 44.0% were normal, 22.1% were overweight, and the remaining 30.0% were obese. Eighty-five patients (30.3%) had a shift to higher BMI categories of overweight/obese, 24 patients (8.6%) changed from being underweight to normal, 93 (33.2%) had maintained their BMI categories, and 78 (27.6%) had decreased in their BMI categories.

### Incidence of hypertension at breast cancer diagnosis and at study entry

At breast cancer diagnosis, nine patients (3.2%) were on anti-hypertensive medications. The median SBP and DBP were 125 (range: 90–172) and 73 mmHg (range: 42–104) respectively. Sixty-two (22.1%) were hypertensive; 38 (13.6%) had raised SBP, nine (3.2%) had raised DBP and 15 (5.3%) had raised SBP and DBP.

At study entry, 17 patients were on anti-hypertensive treatment (6.1%). The median SBP and DBP were 127 (range: 87–206) and 77 mmHg (range: 47–128) respectively. Eighty-six (30.7%) were hypertensive; 37 (13.2%) had raised SBP, 11 (3.9%) had raised DBP and 38 (13.6%) had raised SBP and DBP.

### Lipid profiles at study entry

Two hundred and seventy-one patients had serum fasting lipids determined. Ninety-three patients (34.3%) had high total-cholesterol, 152 (56.1%) had high LDL-cholesterol, 18 (6.6%) had low HDL-cholesterol and 62 (22.9%) had hypertriglyceridaemia.

### Analysis for risk factors associated with weight gain, hypertension and dyslipidaemias after adjuvant chemotherapy

Table 2 illustrates the outcomes of univariate and multivariate analyses on factors associated with weight gain. Univariate analysis revealed that older age at breast cancer diagnosis (OR 0.529, 95% CI 0.365–0.766, *p* = 0.007) as well as older age at study entry (OR 0.593, 95% CI 0.429–0.819, *p* = 0.0015) were both associated with less likelihood of weight gain. However, taxane-containing chemotherapy (OR 1.903, 95% CI 1.073–3.374, *p* = 0.0278), longer duration of adjuvant chemotherapy (OR 1.904, 95% CI 1.137–3.189, *p* = 0.0143), and having experienced CRA (OR 2.375, 95% CI 1.034–5.435, *p* = 0.414) were associated with weight gain. On multivariate analysis, taxane-containing chemotherapy (OR 2.041, 95% CI 1.127–3.696, *p* = 0.0186), having experienced CRA (OR 3.472, 95% CI 1.395–8.621, *p* = 0.0074) and age at breast cancer diagnosis (OR for older patients 0.459, *p* = 0.0001) were independent factors for weight gain.

**Table 2:** Univariate and multivariate analysis on factors associated with weight gain >2%, by stepwise logistic regression

	Univariate analysis			Multivariate analysis		
	OR	95% CI for OR	p	OR	95% CI for OR	*p*
Age at diagnosis	0.529	0.365–0.766	0.0007	0.459	0.309–0.683	0.0001
≤ 35	1	-	-			
36–40	0.767	0.316–1.861	0.5575			
41–45	0.332	0.149–0.741	0.0072			
>/= 1 children before breast cancer diagnosis	0.751	0.428–1.315	0.3160			
1st degree relative with breast cancer	1.425	0.488–4.167	0.5173			
ER positive	0.975	0.566–1.682	0.9282			
PR positive	1.211	0.726–2.022	0.4632			
HER2 over-expression	1.032	0.538–1.981	0.9238			
Received adjuvant radiotherapy	1.352	0.812–2.250	0.2466			
Received adjuvant taxane-containing chemotherapy	1.903	1.073–3.374	0.0278	2.041	1.127–3.696	0.0186
Duration of adjuvant chemotherapy >64 days	1.904	1.137–3.189	0.0143			
Received corticosteroid premedication during chemotherapy	1.605	0.608–4.237	0.3390			
Received adjuvant tamoxifen therapy	1.366	0.778–2.398	0.2781			
Received adjuvant trastuzumab	0.969	0.227–4.140	0.9660			
Use of traditional Chinese medicine since diagnosis	1.303	0.758–2.239	0.3384			
Education	1.299	0.850–1.987	0.2271			
Primary school	1	-	-			
Secondary school	1.447	0.757–2.766	0.2642			
Tertiary school + Higher qualification	1.669	0.718–3.878	0.2336			
Employment- Working	0.954	0.579–1.572	0.8531			
Family income </= HK$ 25,000	1.388	0.832–2.315	0.2097			
Ever Smoker	0.374	0.103–1.357	0.1348			
Ever excessive alcohol intake (>2 units/day)	-	-	0.9868			
Chemotherapy-related amenorrhea	2.375	1.034–5.435	0.0414	3.472	1.395–8.621	0.0074
Post/peri- menopausal at study entry	0.666	0.408–1.085	0.1023			
Age at study entry	0.593	0.429–0.819	0.0015			
≤ 40	1	-				
41–45	0.755	0.297–1.923	0.5561			
46–50	0.467	0.198–1.102	0.0822			
> 50	0.185	0.059–0.580	0.0038			

Table 3 shows the outcomes of univariate and multivariate analyses on factors associated with hypertension. Univariate analysis revealed that estrogen receptor positivity (OR 0.517, 95% CI 0.298–0.895, *p* = 0.0186) and having received adjuvant tamoxifen therapy (OR 0.557, 95% CI 0.313–0.990, *p* = 0.0.0462) were associated with less likelihood of hypertension; while abnormal LDL-cholesterol (OR 1.762, 95% CI 1.027–3.0221, *p* = 0.0396) and overweight/obese at study entry (OR 2.751, 95% CI 1.608–4.707, *p* = 0.0002) were associated with higher likelihood of hypertension. On multivariate analysis, overweight/obese at study entry (OR 2.723, 95% CI 1.568–4.728, *p* = 0.0004) and estrogen receptor positivity (OR 0.552, 95% CI 0.311–0.980, *p* = 0.0423) were independent factors for hypertension.

**Table 3:** Univariate and multivariate analyses on factors associated with hypertension at study entry, by stepwise logistic regression

	Univariate analysis			Multivariate analysis		
	OR	95% CI for OR	*p*	OR	95% CI for OR	*p*
Age at diagnosis	1.243	0.869–1.779	0.2343			
≤ 35	1	-				
36–40	1.000	0.429–2.331	1.0000			
41–45	1.417	0.658–3.050	0.3727			
>/= 1 children before breast cancer diagnosis	1.291	0.715–2.332	0.3695			
1st degree relative with breast cancer	0.926	0.316–2.715	0.8885			
ER positive	0.517	0.298–0.895	0.0186	0.552	0.311–0.980	0.0423
PR positive	0.712	0.419–1.213	0.2115			
HER2 over-expression	1.187	0.610–2.309	0.6134			
Received adjuvant radiotherapy	0.866	0.508–1.477	0.5984			
Received adjuvant taxane-containing chemotherapy	0.887	0.501–1.568	0.6790			
Duration of adjuvant chemotherapy >64 days	0.828	0.483–1.420	0.4928			
Received corticosteroid premedication during chemotherapy	1.046	0.410–2.667	0.9251			
Received adjuvant tamoxifen therapy	0.557	0.313–0.990	0.0462			
Received adjuvant trastuzumab	1.352	0.316–5.789	0.6846			
Use of traditional Chinese medicine since diagnosis	0.947	0.542–1.655	0.8486			
Education	0.789	0.507–1.228	0.2936			
Primary school	1	-	-			
Secondary school	0.907	0.461–1.787	0.7784			
Tertiary school + Higher qualification	0.609	0.246–1.509	0.2840			
Employment- Working	0.815	0.485–1.369	0.4397			
Family income </= HK$ 25,000	0.753	0.441–1.287	0.3000			
Ever Smoker	0.271	0.033–2.198	0.2214			
Ever excessive alcohol intake (>2 units/day)	-	-	0.9877			
Chemotherapy-related amenorrhea	0.778	0.329–1.838	0.5665			
Post/peri- menopausal at study entry	0.930	0.559–1.548	0.7808			
Weight gain >2% at study entry	1.095	0.644–1.864	0.7371			
Total Cholesterol >/= 5.2 mmol/L	1.475	0.865–2.516	0.1534			
LDL Cholesterol >/= 2.6 mmol/L	1.762	1.027–3.021	0.0396			
HDL Cholesterol </= 1.0 mmol/L	1.842	0.700–4.850	0.2160			
Triglyceride >/=1.7 mmol/L	1.568	0.867–2.836	0.1364			
Overweight/obese at study entry	2.751	1.608–4.707	0.0002	2.723	1.568–4.728	0.0004
Age at study entry	1.173	0.852–1.614	0.3273			
≤ 40	1	-				
41–45	1.929	0.738–5.037	0.1799			
46–50	1.774	0.720–4.371	0.2126			
> 50	2.057	0.623–6.794	0.2367			

Hypertension defined as systolic blood pressure >/= 140 mmHg, or diastolic blood pressure >/=90 mmHg

Table 4 shows the factors that were identified by on univariate and multivariate analyses to be associated with dyslipidaemias. Multivariate analysis identified that older age (OR 2.093, 95% CI 1.461–2.997, *p* < 0.0001) and overweight/obese (OR 1.764, 95% CI 1.013–3.073, *p* = 0.0451) were associated with increased likelihood of high total-cholesterol. Use of tamoxifen (OR 0.342, 95% CI 0.179–0.656, *p* = 0.0012), overweight/obese (OR 2.560, 95% CI 1.410–4.649, *p* = 0.0020), older age at study entry (OR 1.625, 95% CI 1.165–2.266, *p* = 0.0042) and having received corticosteroids premedication during chemotherapy (OR 5.747, 95% CI 1.264–26.32, *p* = 0.0236) were associated with high LDL-cholesterol. Becoming post/peri-menopausal (OR 3.629, 95% CI 1.144–11.509, *p* = 0.0286) and overweight/obese at study entry (OR 7.786, 95% CI 1.742–34.795, *p* = 0.0072) were associated with increased likelihood of low HDL-cholesterol. Older age (OR 1.969, 95% CI 1.258–3.017, *p* = 0.0019), taxane-containing chemotherapy (OR 2.552, 95% CI 1.317–4.944, *p* = 0.0055), overweight/obese (OR 2.775, 95% CI 1.446–5.325, *p* = 0.0021) and weight gain (OR 2.213, 95% CI 1.084–4.517, *p* = 0.0292) at study entry were associated with higher likelihood of hypertriglyceridaemia. Factors that were analyzed but were not found to associate with any component of dyslipidaemias on multivariate analysis include: education level; employment and family income statuses; history of smoking and excessive alcohol intake; having child birth before breast cancer diagnosis; family history of first degree relative with breast cancer; breast ER/PR and HER2 status; having received adjuvant radiotherapy, corticosteroid or adjuvant trastuzumab; longer duration of adjuvant chemotherapy; having experienced CRA; and use of traditional Chinese medicine. Additional file 1: Table S1 lists the outcomes of univariate analyses on potential factors in association with abnormal total cholesterol, LDL-cholesterol, HDL-cholesterol ad triglyceride levels.

**Table 4:** Factors identified to be associated with dyslipidaemia based on multivariate analysis, by stepwise logistic regression

	Univariate analysis			Multivariate analysis		
	OR	95% CI for OR	*p*	OR	95% CI for OR	*P*
Total cholesterol: high (*n* = 93) vs normal (*n* = 178)						
Overweight/obese at study entry	1.760	1.032–3.003	0.0379	1.764	1.013–3.073	0.0451
Age at study entry	2.101	1.467–3.010	<0.0001	2.093	1.461–2.997	<0.0001
≤ 40	1	-				
41–45	1.490	0.494–4.489	0.4789			
46–50	3.838	1.397–10.548	0.0091			
> 50	7.020	1.989–24.772	0.0025			
LDL cholesterol: high (*n* = 152) vs normal (*n* = 115)						
Received corticosteroid premedication during chemotherapy	7.576	1.724–33.33	0.0074	5.747	1.264–26.32	0.0236
Received adjuvant tamoxifen	0.388	0.209–0.720	0.0027	0.342	0.179–0.656	0.0012
Overweight/obese at study entry	2.468	1.407–4.330	0.0016	2.560	1.410–4.649	0.0020
Age at study entry	1.626	1.192–2.217	0.0021	1.625	1.165–2.266	0.0042
≤ 40	1	-				
41–45	0.963	0.420–2.212	0.9298			
46–50	1.763	0.813–3.823	0.1510			
> 50	5.383	1.493–19.407	0.0101			
HDL cholesterol: low (*n* = 18) vs. normal (*n* = 251)						
Ever smoker	4.357	0.836–22.707	0.0806	7.772	1.102–54.827	0.0397
Post/peri- menopausal at study entry	3.881	1.243–12.114	0.0196	3.629	1.144–11.509	0.0286
Overweight/obese at study entry	8.193	1.845–36.372	0.0057	7.786	1.742–34.795	0.0072
Triglyceride: high (*n* = 62) vs. normal (*n* = 209)						
Adjuvant taxane-containing chemotherapy	2.040	1.124–3.704	0.0192	2.552	1.317–4.944	0.0055
Weight gain >2%	2.258	1.171–4.355	0.0150	2.213	1.084–4.517	0.0292
Overweight/obese at study entry	2.306	1.282–4.149	0.0053	2.775	1.446–5.325	0.0021
Age at study entry	1.641	1.118–2.410	0.0114	1.969	1.285–3.017	0.0019
≤ 40	1	-				
41–45	1.355	0.401–4.573	0.6246			
46–50	2.558	0.842–7.773	0.0978			
> 50	3.733	0.964–14.461	0.0566			

Definitions of abnormal lipids: total cholesterol >/=5.2 mmol/L; LDL cholesterol >/= 2.6 mmol/L; HDL cholesterol </= 1.0 mmol/L; triglyceride >1.7 mmol/L

## Discussion

Until recently, clinical follow-up of cancer survivors have mainly focused on survival and early detection of cancer recurrence. The advancement in the use of adjuvant therapies has improved outcomes of breast cancer patients, and as a result longer survivals are expected. With increased survival, it is anticipated that long-term toxicities associated with cancer treatments may become more evident; these include effects on physical morbidities and psychosocial symptoms. To our knowledge, the present study is one of the first to assess cardiovascular risk profiles of young premenopausal Chinese patients who had received modern-day adjuvant chemotherapy; apart from body weight and BMI alterations, changes on blood pressure and fasting lipids measurements have also been assessed. As the incidence of breast cancer among young Asian females has been noted to be rapidly increasing, information on these aspects among the studied population is of particular interest [22].

Although the cause of weight gaining after breast cancer diagnosis is unclear, it has been suggested that there may be ethnic differences. In the WHEL study [9], Asian-Americans were found to have a lower risk of gaining weight after chemotherapy than other races. To date, only a few studies have addressed the issue on weight changes in Asian patients. In a study from Shanghai China, breast cancer patients who received adjuvant chemotherapy had mean weight gains of 1.0, 2.0 and 1.0 kg at 6, 18 and 36 months after diagnosis. However, details of the type and duration of chemotherapy were not described [10]. A Korean study involved 195 pre- and post-menopausal patients who received adjuvant chemotherapy (mainly anthracyclines and taxanes regimens) with/without endocrine therapy [23]. Although 10% of the studied population gained weight by >5% at 1 year, the mean weight change was in fact, at a loss of 0.34 kg, indicating that the majority of Korean breast cancer patients did not gain weight after adjuvant treatment. In a third study [24], 98 pre- and post-menopausal Chinese breast cancer patients underwent weight assessment prior to and immediately after having received doxorubicin and/or taxane-containing adjuvant chemotherapy; the results revealed that they had an overall mean weight loss of 0.4 kg. The present study assessed only young premenopausal Chinese patients with a protracted interval of 5 years after adjuvant chemotherapy, 63% had gained weight by >2% and the average gain was 1.8 kg; although this figure appeared to be small, one cannot overlook the proportion of patients who were detected to have abnormal BMI, as this had increased by nearly 60%, from 33% at breast cancer diagnosis to 52% at study entry. The figure is worrisome; according to the World Health Organization (WHO) criteria, significant health risks is associated with obesity, which has been defined as a body mass index (BMI) of 25.0 and over for Asian population [19].

A number of factors have been suggested to lead to weight gain and obesity after adjuvant chemotherapy. These include hyperphagia, younger age, menopausal status, reduced physical activity, longer duration of adjuvant chemotherapy, use of corticosteroids premedication during chemotherapy and pre-treatment body weight [7, 8, 11, 24–28]. The present study has confirmed that weight gain is more common among premenopausal patients who were younger at diagnosis and who experienced CRA; in addition, those who had prior taxane-containing regimen were also at higher risk. The latter could be associated with the use of corticosteroids during chemotherapy, as had been suggested by the study reported by Goodwin et al. [11] Dexamethasone is given as standard premedication prior to taxane therapy to avoid hypersensitivity reactions, and is also part of the optimal antiemetic regimen offered for patients on anthracycline-containing chemotherapy.

Only a few studies have addressed dyslipidaemias after adjuvant chemotherapy in breast cancer patients. In a recent report on Italian pre- and post-menopausal patients with early breast cancer, increase in cholesterol was found in patients who received chemotherapy only, but not in those who also received endocrine therapy [6]. The occurrence of abnormal total- and LDL-cholesterol among premenopausal patients has been attributed to chemotherapy-induced ovarian dysfunction [29–31]. The observation from a recent study lends support to this by showing that peri-menopausal patients undergoing taxane-based adjuvant chemotherapy had the largest change in lipids profiles when compared to pre- or post-menopausal patients, implicating that transition in hormone status may play a role [32]. Tamoxifen has been well-reported to lower total- and LDL-cholesterol [33–36] as early as 3 months after treatment initiation [34, 35]. Further, the administration of adjuvant tamoxifen after chemotherapy has been observed to reverse dyslipidaemias in premenopausal patients [30]. In the present study, a significant proportion of patients were found to have dyslipidaemias after chemotherapy. While tamoxifen was associated with reduced risk of abnormal LDL-cholesterol, increased likelihood of dyslipidaemias was associated with older age, post/peri-menopausal status after chemotherapy, weight gain or overweight/obese, and having received taxane-containing chemotherapy or corticosteroids premedication during adjuvant chemotherapy.

The present study has a few limitations; there is a lack of data on energy expenditures (e.g., physical activity levels, basal metabolic rates), caloric intakes and body composition. Further, blood pressure measurement was only performed once during clinic visit; none-the-less, this provides a glimpse on the cardiovascular status of the studied population. Dyslipidaemia may contribute to adverse cardiovascular outcomes. In this study, over 30% of the patients were detected to be hypertensive. Multivariate analysis on risk of hypertension showed that estrogen receptor positivity was associated with decreased likelihood while overweight/obese was associated with increased likelihood of raised blood pressure. It has to be noted that additional 2 factors were identified on univariate analysis- use of tamoxifen reduced (OR 0.557, 95% CI 0.313–0.990, *p* = 0.0462) while high LDL-cholesterol (OR 1.762, 95% CI 1.027–3.021, *p* = 0.0396) increased the likelihood of hypertension (Additional file 1: Table S1); these factors may have intertwining effects.

It has to be noted that long term toxicities of chemotherapy are not confined to menstrual disturbances, weight and BMI changes. The occurrence of hypertension and dyslipidaemias based on findings from the current study needs to be confirmed with further studies. Studies on bone health have reported that women with premature ovarian failure after adjuvant chemotherapy have increased risk for bone loss when compared to their counterparts who remain premenopausal [37, 38]. Although cognitive dysfunction among patients who developed menopause after chemotherapy has not been confirmed in earlier studies [39, 40], further assessment with improved methodology is required. Moreover, it has been shown that over 30% of cancer survivors suffer from fatigue and sleep disturbances, anxiety, depression and impairment of physical functioning affecting their quality of life [41, 42].

## Conclusions

In summary, the present study suggests that younger premenopausal patients and those who experienced CRA were more susceptible to weight gain and abnormal BMI, which in turn were associated with adverse effects on cardiovascular profiles with dyslipidemias and hypertension. Several reports have suggested that weight gain and obesity affect breast cancer outcomes [43, 44], and a recent report has implicated that high LDL-cholesterol at diagnosis [45] may also have prognostic importance. At the same time, weight gain and obesity not only affect the physical wellbeing of a patient, but have been associated with impaired quality of life among breast cancer patients after adjuvant therapy [46]. These provide good justifications for encouraging lifestyle changes to patients after breast cancer diagnosis [6, 23, 47]. In many developing countries, educational level and economical status are the major determinants of reaching healthcare facilities and preventive interventions. The lack of weight gain in Korean patients after chemotherapy has been attributed in part to patient education, which encouraged patients to maintain healthy body weight [23]. As such, data on interventional studies with lifestyle modifications to control weight and dyslipidaemia are valuable and may provide information to health care providers to better manage patients for impacts of long-term sequelae of anti-cancer treatments.

## Additional file

Additional file 1: Table S1. Univariate and multivariate analysis on factors associated with abnormal total cholesterol, LDL-cholesterol, HDL-cholesterol and triglyceride; by stepwise logistic regression. Outcome of univariate and multivariate analysis on factors associated with abnormal total cholesterol, LDL-cholesterol, HDL-cholesterol and triglyceride. (DOCX 36 kb)

## Abbreviations

BMI: Body mass index
CRA: Chemotherapy-related amenorrhea
DBP: Diastolic blood pressure
HDL: High density lipoprotein
LDL: Low density lipoprotein
LMP: Last menstrual period
SBP: Systolic blood pressure
WHO: World Health Organization
