# Supplementary material for: Profiles of lipids, blood pressure and weight changes among premenopausal Chinese breast cancer patients after adjuvant chemotherapy
Source: BMC Womens Health. 2017 Jul 27;17:55. doi: 10.1186/s12905-017-0409-8 (PMC5532788; doi:10.1186/s12905-017-0409-8)
Supplement: Additional file 1: Table S1. — Univariate and multivariate analysis on factors associated with abnormal total cholesterol, LDL-cholesterol, HDL-cholesterol and triglyceride; by stepwise logistic regression. Outcome of univariate and multivariate analysis on factors associated with abnormal total cholesterol, LDL-cholesterol, HDL-cholesterol and triglyceride. (DOCX 36 kb) [file 12905_2017_409_MOESM1_ESM.docx]

**Table S1. Univariate and multivariate analysis on factors associated with abnormal total cholesterol, LDL-cholesterol, HDL-cholesterol and triglyceride; by stepwise logistic regression**

|  | **Abnormal total cholesterol** | | | **Abnormal LDL-cholesterol** | | | **Abnormal HDL-cholesterol** | | | **Abnormal triglyceride** | | |
| --- | --- | --- | --- | --- | --- | --- | --- | --- | --- | --- | --- | --- |
|  | **Odd Ratio (OR)** | **95% CI for OR** | **p** | **Odd Ratio (OR)** | **95% CI for OR** | **p** | **Odd Ratio (OR)** | **95% CI for OR** | **p** | **Odd Ratio (OR)** | **95% CI for OR** | **p** |
| Age at diagnosis  ≤35  36-40  41-45 | 1.359  1  1.294  1.808 | 0.949-1.946  -  0.548-3.057  0.821-3.984 | 0.0946  0.5563  0.1417 | 0.913  1  0.809  0.795 | 0.654-1.273  -  0.369-1.774  0.387-1.636 | 0.5899  0.5964  0.5340 | 1.566  1  2.568  3.281 | 0.732-3.347  -  0.290-22.767  0.413-26.032 | 0.2477  0.3971  0.2609 | 1.565  1  0.546  1.593 | 1.017-2.409  -  0.197-1.515  0.679-3.739 | 0.0416  0.2451  0.2843 |
| >/= 1 children before breast cancer diagnosis | 2.106 | 1.141-3.887 | 0.0172 | 1.474 | 0.860-2.527 | 0.1579 | 1.972 | 0.554-7.020 | 0.2945 | 2.295 | 0.097-4.801 | 0.0273 |
| 1^st^ degree relative with breast cancer | 1.159 | 0.408-3.293 | 0.7824 | 1.716 | 0.579-5.084 | 0.3298 | 0.925 | 0.115-7.432 | 0.9419 | 0.464 | 0.103-2.101 | 0.3192 |
| ER positive | 0.815 | 0.470-1.412 | 0.4653 | 0.442 | 0.249-0.783 | 0.0051 | 0.773 | 0.279-2.141 | 0.6208 | 1.185 | 0.623-2.252 | 0.6045 |
| PR positive | 0.761 | 0.450-1.286 | 0.3075 | 0.445 | 0.260-0.764 | 0.0033 | 0.607 | 0.231-1.594 | 0.3105 | 1.312 | 0.707-2.433 | 0.3888 |
| HER2 over-expression | 0.949 | 0.482-1.869 | 0.8797 | 0.798 | 0.417-1.527 | 0.4955 | 2.719 | 0.963-7.674 | 0.0589 | 1.280 | 0.616-2.660 | 0.5083 |
| Received adjuvant radiotherapy | 1.180 | 0.690-2.017 | 0.5461 | 1.165 | 0.699-1.942 | 0.5590 | 0.618 | 0.235-1.623 | 0.3282 | 0.747 | 0.415-1.345 | 0.3309 |
| Received adjuvant taxane-containing chemotherapy | 0.820 | 0.466-1.443 | 0.4919 | 0.699 | 0.411-1.191 | 0.1880 | 1.268 | 0.458-3.508 | 0.6478 | 2.040 | 1.124-3.704 | 0.0192 |
| Duration of adjuvant chemotherapy >64 days | 1.151 | 0.669-1.979 | 0.6111 | 1.412 | 0.842-2.369 | 0.1904 | 1.729 | 0.552-5.417 | 0.3472 | 1.333 | 0.712-2.497 | 0.3693 |
| Received corticosteroid premedication during chemotherapy | 0.491 | 0.204-1.180 | 0.1117 | 7.576 | 1.724-33.33 | 0.0074 | 0.238 | 0.070-0.808 | 0.0213 | 0.485 | 0.193-1.215 | 0.1225 |
| Received adjuvant tamoxifen therapy | 0.686 | 0.387-1.215 | 0.1959 | 0.388 | 0.209-0.720 | 0.0027 | 0.817 | 0.280-2.384 | 0.7111 | 1.281 | 0.645-2.544 | 0.4801 |
| Received adjuvant trastuzumab | 1.153 | 0.269-4.936 | 0.8475 | 1.270 | 0.297-5.426 | 0.7472 | - | - | 0.9828 | 1.128 | 0.222-5.733 | 0.8848 |
| Use of traditional Chinese medicine since diagnosis | 0.588 | 0.330-1.048 | 0.0719 | 0.623 | 0.368-1.056 | 0.0785 | 0.658 | 0.210-2.064 | 0.4729 | 0.788 | 0.415-1.495 | 0.4660 |
| Education  Primary school  Secondary school  Tertiary school+ Higher qualification | 0.876  1  0.934  0.766 | 0.566-1.355  -  0.470-1.856  0.318-1.843 | 0.5511  -  0.8461  0.5513 | 0.849  1  0.951  0.724 | 0.557-1.293  -  0.486-1.859  0.313-1.674 | 0.4453  -  0.8827  0.4498 | 0.396  1  0.278  0.246 | 0.169-0.928  -  0.097-0.795  0.048-1.257 | 0.0331  -  0.0169  0.0919 | 0.663  1  0.608  0.451 | 0.403-1.092  -  0.294-1.255  0.167-1.216 | 0.1068  -  0.1785  0.1157 |
| Employment- Working | 0.892 | 0.533-1.492 | 0.6631 | 0.827 | 0.501-1.364 | 0.4559 | 0.598 | 0.226-1.535 | 0.2786 | 0.572 | 0.323-1.016 | 0.0566 |
| Family income </= HK$ 25,000 | 0.932 | 0.555-1.567 | 0.7910 | 1.223 | 0.739-2.022 | 0.4332 | 0.835 | 0.303-2.299 | 0.7273 | 0.738 | 0.405-1.345 | 0.3204 |
| Ever smoker | 2.471 | 0.647-9.432 | 0.1857 | 6.333 | 0.781-51.375 | 0.0839 | 4.357 | 0.836-22.707 | 0.0806 | 0.962 | 0.195-4.754 | 0.9624 |
| Ever excessive alcohol intake (>2 units/day) | - | - | 0.9863 | - | - | 0.9862 | - | - | 0.9939 | - | - | 0.9892 |
| Chemotherapy-related amenorrhea | 1.164 | 0.489-2.772 | 0.7310 | 1.290 | 0.543-3.061 | 0.5642 | 1.301 | 0.281-6.032 | 0.7363 | 1.301 | 0.281-6.032 | 0.7363 |
| Post/peri- menopausal at study entry | 1.940 | 1.165-3.231 | 0.0109 | 1.160 | 0.714-1.884 | 0.5492 | 3.881 | 1.243-12.114 | 0.0196 | 1.438 | 0.567-3.643 | 0.4441 |
| Weight gain >2% at study entry | 1.179 | 0.695-1.998 | 0.5416 | 1.192 | 0.720-1.972 | 0.4948 | 2.025 | 0.647-6.335 | 0.2252 | 2.258 | 1.171-4.355 | 0.0150 |
| Overweight/obese at study entry | 1.760 | 1.032-3.003 | 0.0379 | 2.468 | 1.407-4.330 | 0.0016 | 8.193 | 1.845-36.372 | 0.0057 | 2.306 | 1.282-4.149 | 0.0053 |
| Age at study entry  ≤ 40  41-45  46-50  > 50 | 2.101  1  1.490  3.838  7.020 | 1.467-3.010  -  0.494-4.489  1.397-10.548  1.989-24.772 | <0.0001  0.4789  0.0091  0.0025 | 1.626  1  0.963  1.763  5.383 | 1.192-2.217  -  0.420-2.212  0.813-3.823  1.493-19.407 | 0.0021  0.9298  0.1510  0.0101 | 1.591  1  0.861  3.444  1.409 | 0.833-3.038  -  0.075-9.851  0.436-27.200  0.084-23.762 | 0.1595  0.9043  0.2408  0.8119 | 1.641  1  1.355  2.558  3.733 | 1.118-2.410  -  0.401-4.573  0.842-7.773  0.964-14.461 | 0.0114  0.6246  0.0978  0.0566 |
